# Intrinsic activated thrombin generation for treatment efficacy and monitoring of octocog alfa and emicizumab in severe hemophilia A

**DOI:** 10.1016/j.rpth.2026.106627

**Published:** 2026-04-30

**Authors:** Emma H. Urlings, Floor C.J.I. Heubel-Moenen, René van Oerle, Dave L.S. Hellenbrand, Paola E.J. van der Meijden, Tilman M. Hackeng, Hugo ten Cate, Magdolna Nagy, Yvonne M.C. Henskens, Henri M.H. Spronk

**Affiliations:** 1 Internal Medicine, Maastricht University Medical Center+, Maastricht, The Netherlands; 2 Cardiovascular Research Institute Maastricht (CARIM), Maastricht University, Maastricht, The Netherlands; 3 Biochemistry, Maastricht University, Maastricht, The Netherlands; 4 Coagulation Profile B.V., Maastricht, The Netherlands; 5 Central Diagnostic Laboratory, Maastricht University Medical Center+, Maastricht, The Netherlands

**Keywords:** thrombin generation assay, hemophilia A, emicizumab, monitoring, intrinsic coagulation pathway

## Abstract

**Background:**

Monitoring patients with severe hemophilia A (SHA) receiving nonfactor replacement therapies such as emicizumab presents significant challenges, because conventional activated partial thromboplastin time-based assays are subject to interference from these treatments. Therefore, we hypothesized that an intrinsic activated thrombin generation assay (TGA) could be an improved alternative.

**Objectives:**

To investigate the sensitivity and applicability of intrinsic activated TGA compared with extrinsic activated assays for monitoring SHA patients on factor (F)VIII (FVIII) and non-FVIII replacement products.

**Methods:**

We developed a novel intrinsic trigger reagent (platelet-poor plasma [PPP] Reagent INT) and compared it with tissue factor (TF) reagents, as well as an FXIa-based reagent, using the Calibrated Automated Thrombography method. Thrombin generation was measured in deficient plasmas, healthy volunteers (*n* = 103), FVIII-deficient plasma spiked with octocog alfa or emicizumab, and plasma from SHA patients on emicizumab (*n* = 22).

**Results:**

PPP Reagent INT specifically activated the intrinsic pathway, showing minimal thrombin generation in FXII- and FXI-deficient plasma, and no thrombin generation in FIX- and FVIII-deficient plasma, whereas activation occurred independently of prekallikrein. Linear regression analysis showed superior sensitivity for PPP Reagent INT triggered thrombin generation in FVIII-deficient plasma spiked with octocog alfa or emicizumab. In plasma from SHA patients, PPP Reagent INT demonstrated sixfold and fourfold greater sensitivity to emicizumab level changes compared with TF-based reagents.

**Conclusion:**

Intrinsic activated TGA using PPP Reagent INT provides enhanced sensitivity for monitoring SHA patients on both FVIII and non-FVIII replacement products compared with TF-based triggers, representing a promising tool for personalized monitoring.

## Essentials

• Monitoring patients with SHA on treatments such as emicizumab is challenging.
• The intrinsic pathway TGA reagent PPP Reagent INT was developed.
• PPP Reagent INT was sixfold more sensitive to emicizumab than tissue-factor-based reagents.
• PPP Reagent INT is a promising tool for personalized monitoring of SHA patients on therapy.

## 1 Introduction

Hemophilia A is a bleeding disorder caused by a deficiency of the clotting factor (F)VIII (FVIII) due to defects in the gene encoding that factor [1,2]. Patients with severe hemophilia A (SHA) are traditionally monitored using activated partial thromboplastin time (aPTT) clotting assays. However, many novel treatments interfere with these assays [3]. An example of such a treatment is emicizumab (Hemlibra), a bispecific antibody that mimics the function of FVIII by binding activated FIX (FIXa) and FX (FX), thus activating FX [2]. Frequent monitoring of emicizumab levels is not required because the plasma concentration of emicizumab remains stable when the maintenance doses are taken consistently [2]. However, exceptions may occur in emergency situations, in the perioperative period, after switching or adding another factor replacement therapy, and in the presence of anti-FVIII alloantibodies. Moreover, given the high costs associated with emicizumab treatment, recent studies have explored the potential for dose reduction [4,5]. A Dutch pharmacokinetic-guided dose reduction study demonstrated that targeting emicizumab concentrations of 30 ± 5 μg/mL, representing an average dose reduction of 39%, was noninferior to conventional dosing in preventing bleeding episodes [5]. This finding underscores the clinical relevance of individualized emicizumab monitoring [4].

Despite this clinical need to monitor emicizumab treatment, available laboratory assays are considered suboptimal. For instance, the presence of emicizumab in plasma shortens the aPTT, leading to an overestimation of the FVIII concentration, while a discrepancy exists between human- and bovine-based chromogenic FVIII assays [2,6]. Furthermore, the available methods do not adequately reflect the physiological *in vivo* hemostatic balance. The aPTT and prothrombin time clotting assays are based on the time until the first fibrin formation, thereby capturing ∼5% of the total thrombin generated. Additionally, the anticoagulant drivers are not accounted for in the prothrombin time and aPTT [7]. The thrombin generation assay (TGA) offers an alternative to these assays and enables the overall assessment of the plasma coagulation system.

Coagulation and, thus, thrombin generation can be activated via either the extrinsic (tissue factor [TF] triggered) or intrinsic pathway. Although extrinsic activated TGA using TF is widely employed, it has notable limitations in accurately reflecting the hemostatic activity of agents acting in the intrinsic pathway. Since emicizumab functions as an activated FVIII mimetic within the intrinsic pathway, an intrinsic-triggered TGA may provide more physiologically relevant assessment of its hemostatic effect than conventional TF-based methods. Additionally, the relationship between emicizumab concentrations and TGA parameters has not been conclusively established. Given the limitations of the classical coagulation assays and the potential increased sensitivity of a global assay, we developed and validated a novel intrinsic-triggered TGA to better reflect the hemostatic potential of SHA patients receiving emicizumab treatment.

## 2 METHODS

### 2.1 Healthy donors and SHA patients on emicizumab

Blood was collected from 103 healthy volunteers and 22 SHA patients on emicizumab after all patients provided written informed consent. The study was approved by the local medical ethical committee of the MUMC+ in accordance with the Declaration of Helsinki. Data from SHA patients on emicizumab were derived from plasma samples obtained as leftover material from routine clinical monitoring. The use of this material was permitted in accordance with the Dutch Code of Conduct for Health Research (Gedragscode Gezondheidsonderzoek 2022). Healthy volunteers were recruited among employees and students from the central diagnostic laboratory of the study center as part of the establishment and verification of reference intervals for health-associated laboratory tests (METC152015, NL52640.068.15).

Emicizumab levels in plasma from SHA patients were determined using a modified one-stage assay with Siemens reagents on a Sysmex Coagulation System-2500 (CS-2500; Siemens Healthineers) and calibrated using Biophen Emicizumab Calibrator (R2 Diagnostics) [6].

### 2.2 Plasma

In all participants, venous blood was drawn using 21-gauge vacutainer Eclipse needles (Becton Dickinson) and collected in Vacuette 3.2% (w/v) sodium citrate tubes (Greiner Bio-One). Platelet-poor plasma (PPP) was prepared by first centrifuging the blood samples at 2500 ×*g* for 5 minutes before removing the plasma and centrifuging again at 10,000 ×*g* for 10 minutes [8]. Both centrifugation steps were performed at room temperature. PPP was snap frozen using liquid nitrogen before storage at −80 °C until later use.

For all experiments, commercially available congenital FXII-, FXI-, FIX- and FVIII-deficient plasma (George King Biomedical), and Prolytix plasma (plasma deficient in FVIII and antithrombin [AT]; HYPHEN Biomed SAS) or institutional normal pooled (NP) plasma were used.

For the spiking of plasmas, emicizumab (Roche Nederland B.V.), octocog alfa (Takeda Nederland B.V.), and AT (HYPHEN Biomed SAS) were used.

### 2.3 Thrombin generation

Thrombin generation was determined using the Calibrated Automated Thrombography method by means of the Fluoroskan Ascent Fluorimeter (Thermo Fisher Scientific) equipped with a 390/460 nm filter using Thrombinoscope 5.0 software (Thrombinoscope B.V.). Each measurement well contained a total volume of 120:80 μL plasma, 20 μL trigger reagent, and 20 μL fluorogenic substrate (2.5 mM Z-Gly-Gly-Arg-AMC, 100 nM CaCl_2_). Each measurement was conducted in duplicate and calibrated against duplicate samples containing the same plasma as the measurement well and 20 μL of calibrator (Thrombinoscope B.V.t). The 96-well plates were incubated for 10 minutes at 37 °C before addition of the fluorogenic substrate. The TGA was activated using the extrinsic trigger reagents PPP Reagent LOW, PPP Reagent, or PPP Reagent HIGH (Stago BNL) containing a low, intermediate, and high TF concentration, respectively; the intrinsic trigger reagent PPP Reagent INT (Coagulation Profile B.V.) containing a contact pathway activator; or through 500 pM FXIa (Innovative Research) in the presence of 50 μg/mL thermostable inhibitor of contact activation (TICA; kind gift from Prof. T. Hackeng).

### 2.4 Statistical analysis

**Figure 1:**
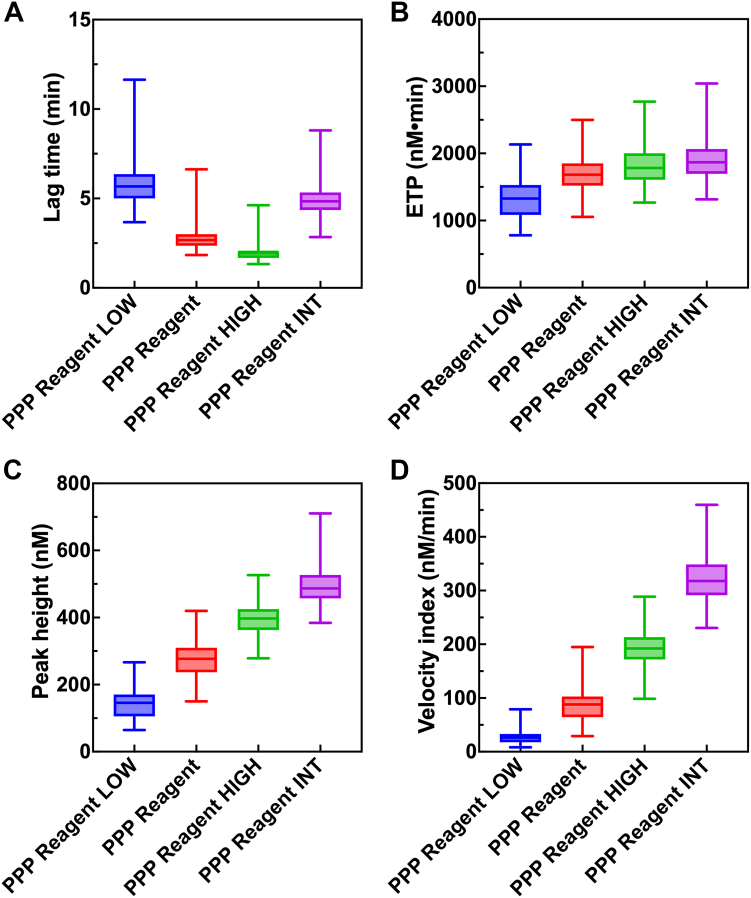
Parameters derived from thrombin generation measured in 103 healthy volunteers using the trigger reagents PPP Reagent LOW, PPP Reagent, PPP Reagent HIGH, and PPP Reagent INT, visualized using the median and IQR (25th-75th percentile). The effect of the different trigger reagents on lag time (A), endogenous thrombin potential (B), peak height (C), and velocity index (D). PPP, platelet-poor plasma.

All statistical analyses and creation of thrombograms were performed using the software GraphPad Prism 10 (GraphPad Software). The reference ranges in healthy volunteers were determined using the 2.5th to 97.5th percentiles. Data are visualized in Figure 1 as boxplots displaying the median and IQR (25th-75th percentile). Simple linear regression analyses were performed using GraphPad Prism 10. The slope was tested against the null hypothesis of zero using an *F*-test. Intra- and inter-assay variability were determined using the CLSI Guideline EP05-A3.

## 3 Results

### 3.1 Effect of PPP Reagent INT on intrinsic activation

**Figure 2:**
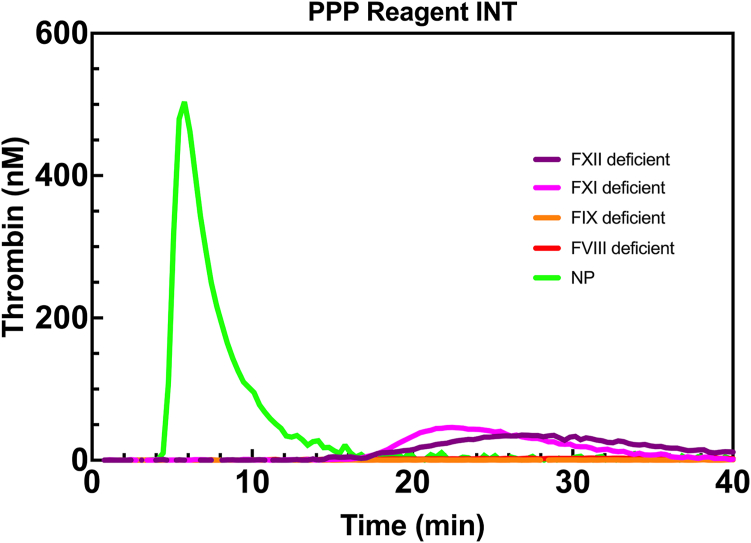
Representative PPP Reagent INT triggered thrombin generation curves obtained from FXII-, FXI-, FIX-, and FVIII-deficient plasma and NP plasma. FVIII, factor VIII; FIX, factor IX; FXI, factor XI; FXII, factor XII; NP, normal pooled; PPP, platelet-poor plasma.

The PPP Reagent LOW, PPP Reagent, PPP Reagent HIGH, and PPP Reagent INT triggered thrombin-generation profiles for FXII-, FXI-, FIX-, or FVIII-deficient plasma were compared with NP. Activation by PPP Reagent INT resulted in minimal thrombin generation in FXII (peak height 37 ± 8 nM) and FXI (peak height 46 ± 0 nM) deficient plasma (Figure 2). No thrombin was generated in FIX- and FVIII-deficient plasma. The resulting thrombograms derived from activation using the extrinsic trigger reagents PPP Reagent LOW, PPP Reagent, and PPP Reagent HIGH are shown in Supplementary Figure 1.

### 3.2 Validation of PPP Reagent INT in plasma derived from healthy volunteers

To determine the inter- and intra-assay coefficient of variances (CVs) of the extrinsic trigger reagents PPP Reagent LOW, PPP Reagent, and PPP Reagent HIGH and intrinsic trigger reagent PPP Reagent INT, thrombin generation in NP was measured in duplicate on six consecutive days. The inter- and intra-assay CVs observed for PPP Reagent INT were within clinically acceptable ranges as defined by CLSI EP05-A3 guidelines and were overall comparable to or lower than those observed for the extrinsic trigger reagents, supporting the reproducibility and reliability of the method (Supplementary Table 1). Internal validation with FVIII-deficient plasma demonstrated comparable CVs to those obtained with NP (data not shown).

Thrombin generation was measured in plasma derived from 103 healthy volunteers using the trigger reagents PPP Reagent LOW, PPP Reagent, PPP Reagent HIGH, and PPP Reagent INT. Subsequently, reference ranges were determined from the derived parameters’ lag time, endogenous thrombin potential, peak height, and velocity index (Supplementary Table 2). The parameters are visualized using boxplots (Figure 1).

### 3.3 Sensitivity of PPP Reagent INT toward (non-)FVIII replacement products

**Figure 3:**
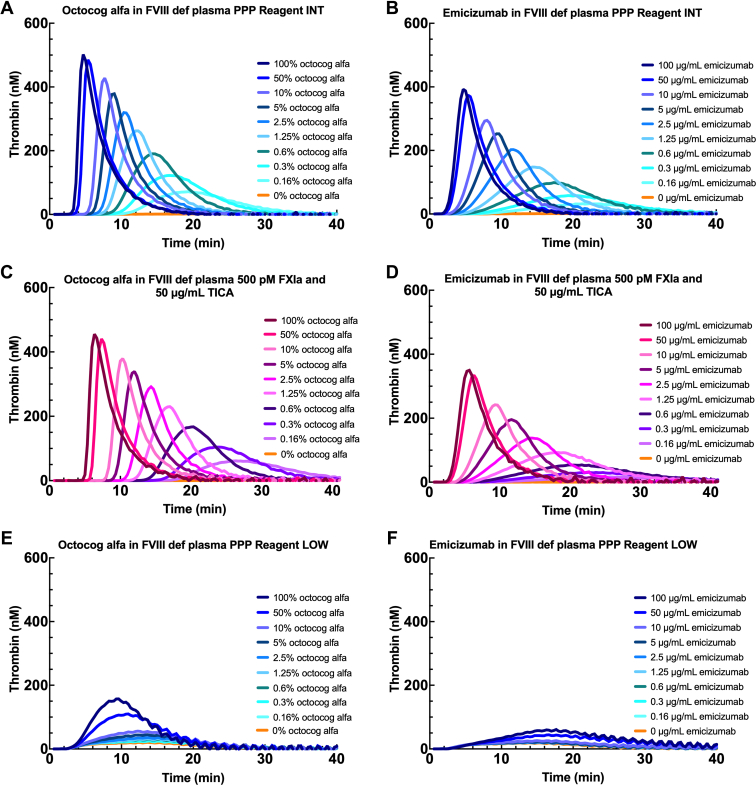
Thrombin generation in FVIII-deficient plasma initiated by PPP Reagent INT, FXIa with TICA, or PPP Reagent LOW. The effect of using PPP Reagent INT on thrombin generation in FVIII-deficient plasma spiked with 0% to 100% octocog alfa (A) or 0 to 100 μg/mL emicizumab (B). The effect of using 500 pM FXIa with 50 μg/mL TICA on thrombin generation of FVIII-deficient plasma spiked with 0% to 100% octocog alfa (C) or 0 to 100 μg/mL emicizumab (D). The effect of using PPP Reagent LOW on thrombin generation of FVIII-deficient plasma spiked with 0% to 100% octocog alfa (E) or 0 to 100 μg/mL emicizumab (F). FVIII, factor VIII; FXIa, activated factor XI; PPP, platelet-poor plasma; TICA, thermostable inhibitor of contact activation.

Next, the sensitivity of PPP Reagent INT toward a FVIII replacement product and the FVIII-mimetic emicizumab was evaluated and compared with FXIa-triggered TG, which has been shown to have increased sensitivity for low levels of FVIII compared with TF-mediated TGA [[9], [10], [11]]. PPP Reagent INT or 500 pM FXIa combined with 50 μg/mL TICA (to inhibit FXIIa-mediated contact activation, as previously described by Van De Berg et al. [12]) was used to trigger thrombin generation in FVIII-deficient plasma spiked with octocog alfa or emicizumab. For both trigger reagents, dose-dependent thrombin generation was observed with each treatment. For both trigger reagents, dose-dependent thrombin generation was observed with both octocog alfa and emicizumab with sensitivity in the lowest range for both octocog alfa (0%-2.5%) and emicizumab (0-2.5 μg/mL, Figure 3A–D). Notably, the extrinsic trigger reagents PPP Reagent LOW, PPP Reagent, and PPP Reagent HIGH showed no distinction between the various concentrations of either FVIII product (Figure 3E–F and Supplementary Figure 2). The lower limit of sensitivity for PPP Reagent INT was determined at 0.16% octocog alfa and 0.16 μg/mL emicizumab, as these were the lowest concentrations tested and were distinguishable from samples containing no octocog alfa or emicizumab.

### 3.4 Correlations of peak height for the different reagents

To assess the sensitivity of TGA to (non-)factor replacement products, thrombin generation was measured in FVIII-deficient plasma spiked with increasing concentrations of either octocog alfa or emicizumab. Thrombin generation was triggered using extrinsic pathway reagents with varying TF concentrations: PPP Reagent LOW, PPP Reagent, and PPP Reagent HIGH, as well as the intrinsic activators PPP Reagent INT and FXIa. Linear regression analysis was performed to evaluate the relationship between replacement product concentration and TGA parameters. The slope of the regression line (β) was used as an indicator of assay sensitivity, with a steeper slope reflecting greater sensitivity to changes in replacement product concentration. The strongest correlation was observed for the parameter peak height, which was therefore explored in more detail.

**Figure 4:**
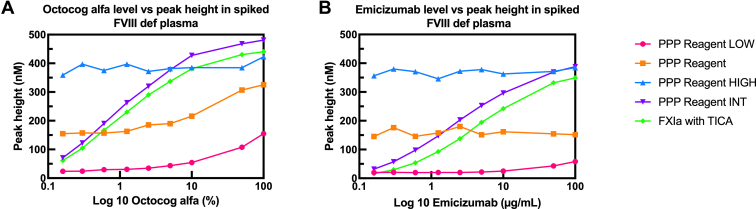
Octocog alfa or emicizumab level plotted against the peak height (derived from thrombin generation measured using PPP Reagent LOW, PPP Reagent, PPP Reagent HIGH, PPP Reagent INT, and FXIa with TICA) in FVIII-deficient plasma spiked with 0% to 100% octocog alfa (A) or with 0 to 100 μg/mL emicizumab (B). FVIII, factor VIII; FXIa, activated factor XI; PPP, platelet-poor plasma; TICA, thermostable inhibitor of contact activation.

Linear regression analysis assessing the sensitivity to octocog alfa concentrations resulted in statistically significant slopes for PPP Reagent LOW (β = 1.32, *P* < .0001), PPP Reagent (β = 1.83, *P* < .0001), PPP Reagent HIGH (β = 0.37, *P* = .0235), PPP Reagent INT (β = 3.36, *P* = .0430), and FXIa with TICA (β = 3.15, *P* = .0367). PPP Reagent INT had the steepest slope, indicating increased sensitivity of this trigger toward changes in the octocog alfa level (Figure 4A).

In addition, linear regression was performed to evaluate the sensitivity for the emicizumab levels. The slopes were not statistically significant for the extrinsic reagents PPP Reagent (β = −0.07, *P* = .5880) and PPP Reagent HIGH (β = 0.17, *P* = .1673). The slopes were statistically significant for PPP Reagent LOW (β = 0.40, *P* < .0001), PPP Reagent INT (β = 3.17, *P* = .0126), and FXIa with TICA (β = 3.16, *P* = .0049). The PPP Reagent INT had the steepest slope, indicating increased sensitivity of this assay toward changes in the emicizumab level (Figure 4B). The octocog alfa or emicizumab level plotted against the other parameters can be found in Supplementary Figures 3 and 4.

### 3.5 Different reagents in plasma derived from SHA patients on emicizumab

**Figure 5:**
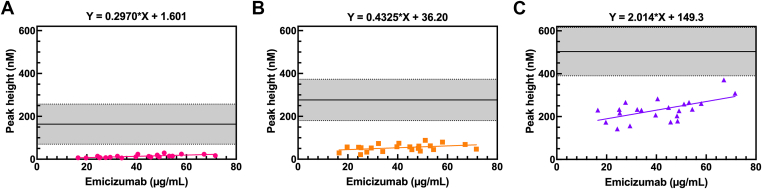
Emicizumab level vs peak height, derived from thrombin generation measured using PPP Reagent LOW (A), PPP Reagent (B), and PPP Reagent INT (C), in plasma from SHA patients on emicizumab. Corresponding reference ranges measured in healthy volunteers are indicated within plotted lines in each graph. PPP, platelet-poor plasma; SHA, severe hemophilia A.

Emicizumab levels and thrombin generation triggered with PPP Reagent LOW, PPP Reagent, and PPP Reagent INT were measured in 22 SHA patients on emicizumab (16.5-71.6 μg/mL) treatment. Linear regression analysis showed that slopes were statistically significant for PPP Reagent LOW, PPP Reagent, and PPP Reagent INT (β = 0.30, *P* < .0001; β = 0.43, *P* = .0094; and β = 2.01, *P* < .0001, respectively). PPP Reagent INT was over sixfold and fourfold more sensitive to changes in the emicizumab level than PPP Reagent LOW and PPP Reagent, respectively. Across all reagents, the peak height values observed in patients receiving emicizumab were lower than those observed in healthy volunteers (Figure 5).

### 3.6 Comparison of the different reagents in combined FVIII and AT deficiency

**Figure 6:**
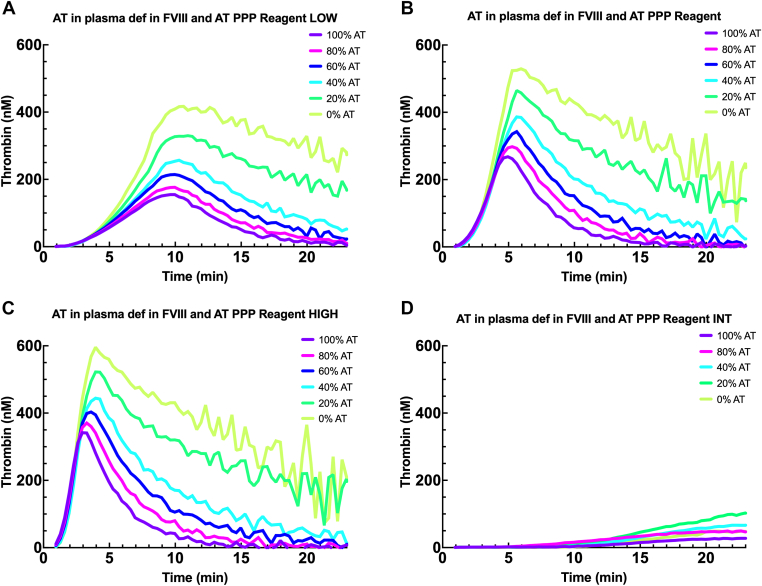
Thrombin generation in plasma deficient in AT and FVIII spiked with AT. The effect of using PPP Reagent LOW (A), PPP RFeagent (B), PPP Reagent HIGH (C), and PPP Reagent INT (D) on thrombin generation of plasma deficient in AT and FVIII spiked with 0% to 100% AT. AT, antithrombin; FVIII, factor VIII; PPP, platelet-poor plasma.

It has been hypothesized that reducing the AT level in hemophilia patients through fitusiran (Sanofi) potentially increased plasma thrombin generation, thereby normalizing hemostasis in SHA patients [13,14]. To mimic thrombin generation in hemophilia A patients on siRNA treatment targeting AT, reagents PPP Reagent LOW, PPP Reagent and PPP Reagent HIGH, and PPP Reagent INT were used as triggers for combined AT and FVIII-deficient plasma spiked with AT. PPP Reagent LOW, PPP Reagent, or PPP Reagent HIGH showed the highest sensitivity for AT levels in FVIII-deficient plasma, whereas PPP Reagent INT hardly provided AT dose-response thrombin generation profiles (Figure 6). This highlights that the choice of reagent should depend on the treatment provided and not solely on the clinical condition.

## 4 Discussion

This study aimed to investigate the applicability of intrinsic activated TGA compared with the extrinsic activated assay to monitor SHA patients on FVIII or non-FVIII. When triggered with PPP Reagent INT, minimal thrombin generation was observed in FXII- and FXI-deficient plasma, while no thrombin was generated in FIX- and FVIII-deficient plasma. These findings confirm that PPP Reagent INT specifically initiates activation via the intrinsic pathway. PPP Reagent INT had the steepest slope in linear regression analysis, indicating that this was the most sensitive reagent for detecting changes in both octocog alfa and emicizumab levels (in spiking experiments as well as in patient samples) among the reagents tested. In plasma from SHA patients, PPP Reagent INT demonstrated sixfold and fourfold greater sensitivity to emicizumab level changes compared with TF-based reagents. The extrinsic trigger reagents PPP Reagent LOW, PPP Reagent, and PPP Reagent HIGH were shown to be more suited in hemophilia patients on treatments targeting AT.

The PPP reagent INT triggered thrombin generation in FVIII-deficient plasma spiked with either octocog alfa or emicizumab, which was comparable to the profiles obtained with FXIa as trigger. van de Berg et al. [15] demonstrated that TGA initiated by TF alone at varying concentrations was unable to differentiate between FVIII levels <20%, whereas the combined low TF/FXIa trigger increased sensitivity for FVIII. Our findings extend these observations by showing that FXIa-triggered TGA (without TF but with added TICA to inhibit FXIIa), maintaining high sensitivity for FVIII replacement products. Although both PPP Reagent INT and FXIa triggers demonstrated similar sensitivity, PPP Reagent INT is more suitable for clinical laboratory practice because it can be standardized (eg, through lyophilization) and is more cost-effective. Importantly, compared with the combined low TF/FXIa trigger, PPP Reagent INT has a higher sensitivity toward FVIII in the low range. Therefore, PPP Reagent INT appears most suitable for the evaluation of (non-)factor replacement therapies targeting the intrinsic pathway. Further studies are required to determine whether emerging non-FVIII replacement therapies other than emicizumab that have potentially greater potency can also be reliably assessed using PPP Reagent INT.

Currently, no validated laboratory assay exists to assess the hemostatic capacity of emicizumab in SHA patients during severe bleeding episodes. Although multiple studies have demonstrated a strong correlation between FVIII levels and TGA parameters, no conclusive evidence has been published regarding the relationship between emicizumab levels and TGA outcomes [16,17]. A recent study by van der Zwet et al. [18] demonstrated no clinically relevant correlations between TG parameters (for both TF and FXIa-triggered TGA) and emicizumab concentration in 49 patients with hemophilia A on steady-state emicizumab prophylaxis. However, in this study, strong correlations were found for the parameter peak height for PPP Reagent LOW, PPP Reagent INT, and FXIa. It should be noted that van der Zwet et al. [18] measured only high emicizumab concentrations (median ∼70 μg/mL), which may limit the ability to detect a concentration-dependent correlation. This is particularly relevant given emerging evidence that lower target emicizumab concentrations may be clinically sufficient. Consequently, assays with enhanced sensitivity across a broader concentration range, including these lower levels, are increasingly important for individualized patient monitoring [4,5]. Another difference between the two studies was the FXIa trigger used. Unlike this study, van der Zwet et al. [18] did not inhibit contact activation through TICA or corn trypsin inhibitor. This is relevant, as FXIa-based triggers without such inhibitors may permit unwanted contact pathway activation, which can artificially increase thrombin generation and thereby mask the true effects of FVIII or emicizumab.

For FVIII replacement products, such as octocog alfa, the target FVIII activity levels required for various physical activities and surgical procedures have been well established in patients with SHA. In contrast, for nonfactor replacement therapies such as emicizumab, no consensus has been reached regarding an equivalent FVIII activity level [19,20]. The TGA has been proposed as a method of achieving this. However, it may be argued that establishing a reliable FVIII equivalence for emicizumab is unfeasible [20]. Emicizumab is fully dependent on the amount of FIXa available and, thus, fully dependent on the TF or FXIa concentrations used to trigger TG [19,20]. This dependency limits the ability to directly correlate emicizumab levels with FVIII activity. Hence, we argue that TGA should be used to personalize and monitor treatment in individual patients. The enhanced sensitivity of PPP Reagent INT to changes in emicizumab levels, particularly at lower concentrations, makes it well suited for this purpose. Further studies are needed to correlate TG parameters with clinical bleeding outcomes, as well as to evaluate TG in relation to other nonfactor replacement therapies beyond emicizumab. A limitation of this study is the absence of longitudinal measurements, which limits the ability to assess intra-individual changes over time and to evaluate the predictive value of TG parameters for future bleeding risk. This study focused on patients with SHA. The performance of PPP Reagent INT in patients with moderate or mild hemophilia A, who have residual FVIII activity of 1% to 5% and 5% to 40%, respectively, was not assessed. Given the potential ceiling effect at higher FVIII activity levels, the clinical utility of this assay in moderate and mild hemophilia warrants further investigation.

## 5 Conclusion

This study provided novel evidence for the applicability of intrinsic activated TGA using PPP Reagent INT for monitoring of SHA patients on both FVIII and the non-FVIII replacement product emicizumab. Compared with TF, intrinsic activation with PPP Reagent INT enhanced sensitivity for octocog alfa or emicizumab in plasma TG. Therefore, intrinsic activated TGA is a promising tool for monitoring of SHA patients on different therapies.
