# Supplementary material for: Intrinsic activated thrombin generation for treatment efficacy and monitoring of octocog alfa and emicizumab in severe hemophilia A
Source: Res Pract Thromb Haemost. 2026 Apr 30;10(4):106627. doi: 10.1016/j.rpth.2026.106627 (PMC13241885; doi:10.1016/j.rpth.2026.106627)
Supplement: Supplementary Tables S1 and S2 [file mmc1.docx]

## Supplemental data


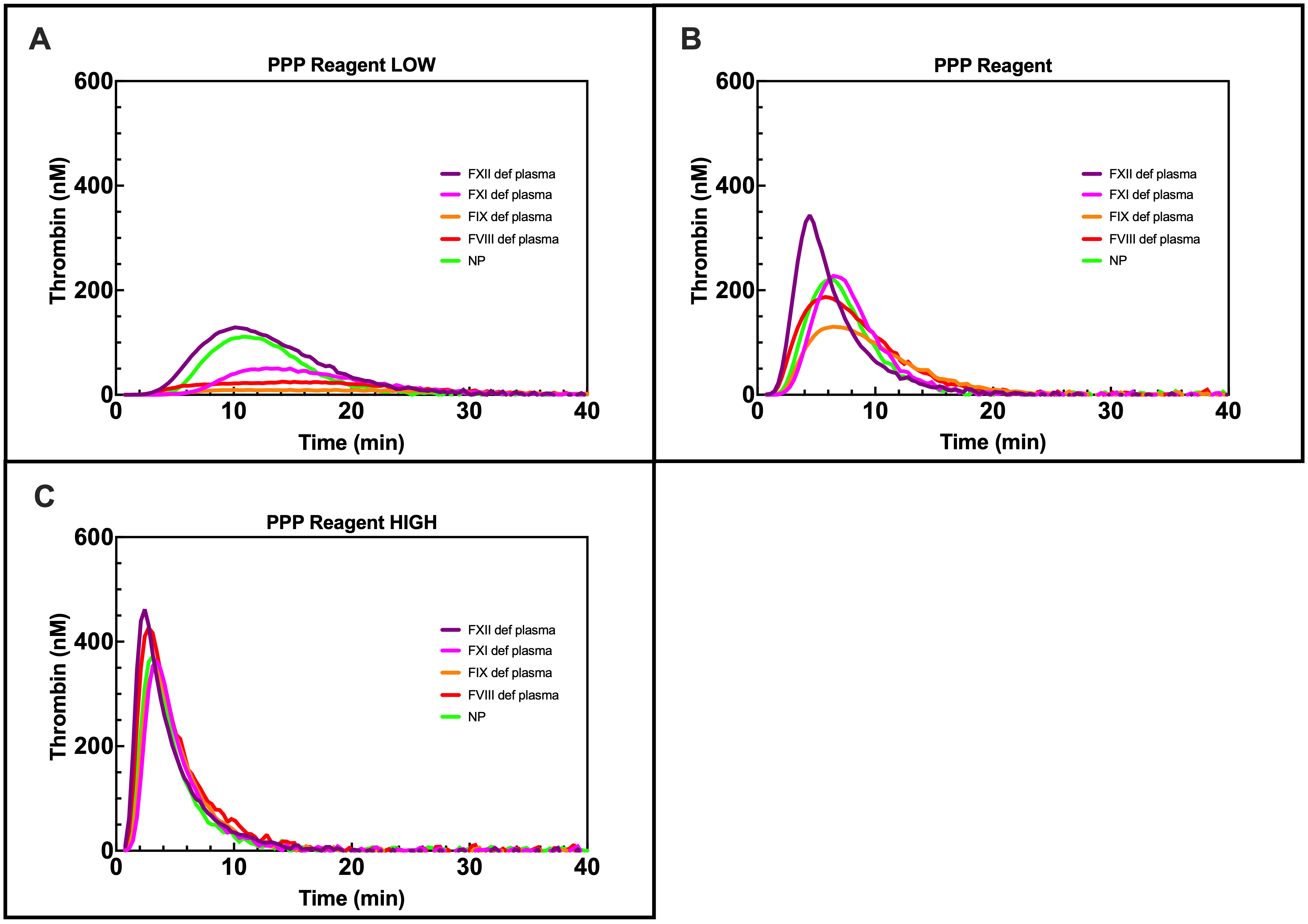


Supplemental figure 1: Thrombin generation initiated by PPP Reagent LOW (A), PPP Reagent (B) and PPP Reagent HIGH (C) and in FXII, FXI, FIX and FVIII deficient plasma, and NP.

Supplemental table 1: Inter- and intra-assay CVs of the trigger reagents PPP Reagent LOW, PPP Reagent, PPP Reagent HIGH and PPP Reagent INT measured in NP in duplicate on 6 consecutive days.

|  | Inter-assay coefficient of variation (%) | | | |
| --- | --- | --- | --- | --- |
| Parameter | **PPP Reagent LOW** | **PPP Reagent** | **PPP Reagent HIGH** | **PPP Reagent INT** |
| Lag time (min) | 3.52 | 3.44 | 5.34 | 4.05 |
| ETP (nM•min) | 6.18 | 3.99 | 5.45 | 2.60 |
| Peak height (nM) | 6.89 | 5.67 | 3.36 | 2.19 |
| Velocity index (nM/min) | 10.00 | 9.63 | 4.06 | 10.02 |
|  | **Intra-assay coefficient of variation (%)** | | | |
| Parameter | **PPP Reagent LOW** | **PPP Reagent** | **PPP Reagent HIGH** | **PPP Reagent INT** |
| Lag time (min) | 2.63 | 1.78 | 0.00 | 3.18 |
| ETP (nM•min) | 4.84 | 3.42 | 4.47 | 2.29 |
| Peak height (nM) | 3.51 | 3.74 | 2.50 | 1.25 |
| Velocity index (nM/min) | 6.40 | 5.50 | 2.87 | 8.20 |

Supplemental table 2: Reference ranges for thrombin generation parameters (lag time, ETP, peak height, and velocity index) measured in plasma from 103 healthy volunteers using PPP Reagent LOW, PPP Reagent, PPP Reagent HIGH, and PPP Reagent INT.

| **Reagent** | **Parameter** | **Reference range (Percentiles 2.5th – 97.5th)** | **[Min-max]** |
| --- | --- | --- | --- |
| **PPP Reagent LOW** | Lag time (min) | 3.78 – 8.01 | [3.67-11.64] |
|  | ETP (nM•min) | 803.7 – 1929 | [781.4-2135] |
|  | Peak height (nM) | 70.0 – 257 | [64.4-267] |
|  | Velocity index (nM/min) | 9.65 – 66.94 | [8.23-79.1] |
| **PPP Reagent** | Lag time (min) | 1.96 – 4.01 | [1.84-6.63] |
|  | ETP (nM•min) | 1230 – 2289 | [1054-2500] |
|  | Peak height (nM) | 180 – 373 | [150-420] |
|  | Velocity index (nM/min) | 33.3 – 168 | [29.1-195] |
| **PPP Reagent HIGH** | Lag time (min) | 1.35 – 2.67 | [1.33-4.62] |
|  | ETP (nM•min) | 1317 – 2437 | [1267-2771] |
|  | Peak height (nM) | 311 – 479 | [278-527] |
|  | Velocity index (nM/min) | 119 – 265 | [98.7-289] |
| **PPP Reagent INT** | Lag time (min) | 3.53 – 6.33 | [2.84-8.81] |
|  | ETP (nM•min) | 1346 – 2557 | [1315-3041] |
|  | Peak height (nM) | 390 – 616 | [384-711] |
|  | Velocity index (nM/min) | 235 – 417 | [230-460] |


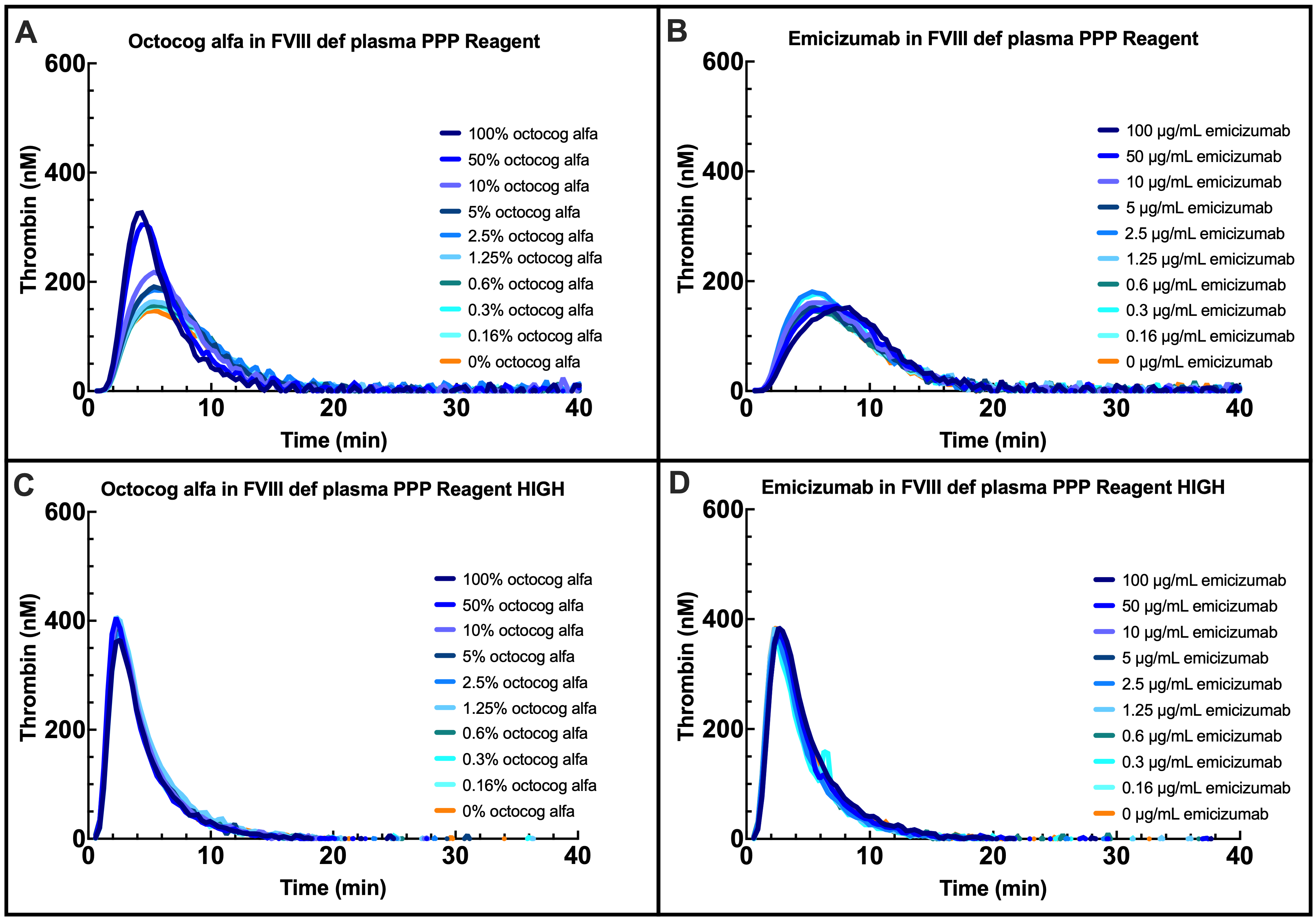


Supplemental figure 2: Thrombin generation in FVIII deficient plasma initiated by PPP Reagent or PPP Reagent HIGH. The effect of using PPP Reagent on thrombin generation in FVIII deficient plasma spiked with 0-100% octocog alfa (A) or 0-100 µg/mL emicizumab (B). The effect of using PPP Reagent HIGH on thrombin generation in FVIII deficient plasma spiked with 0-100% octocog alfa (C) or 0-100 µg/mL emicizumab (D).


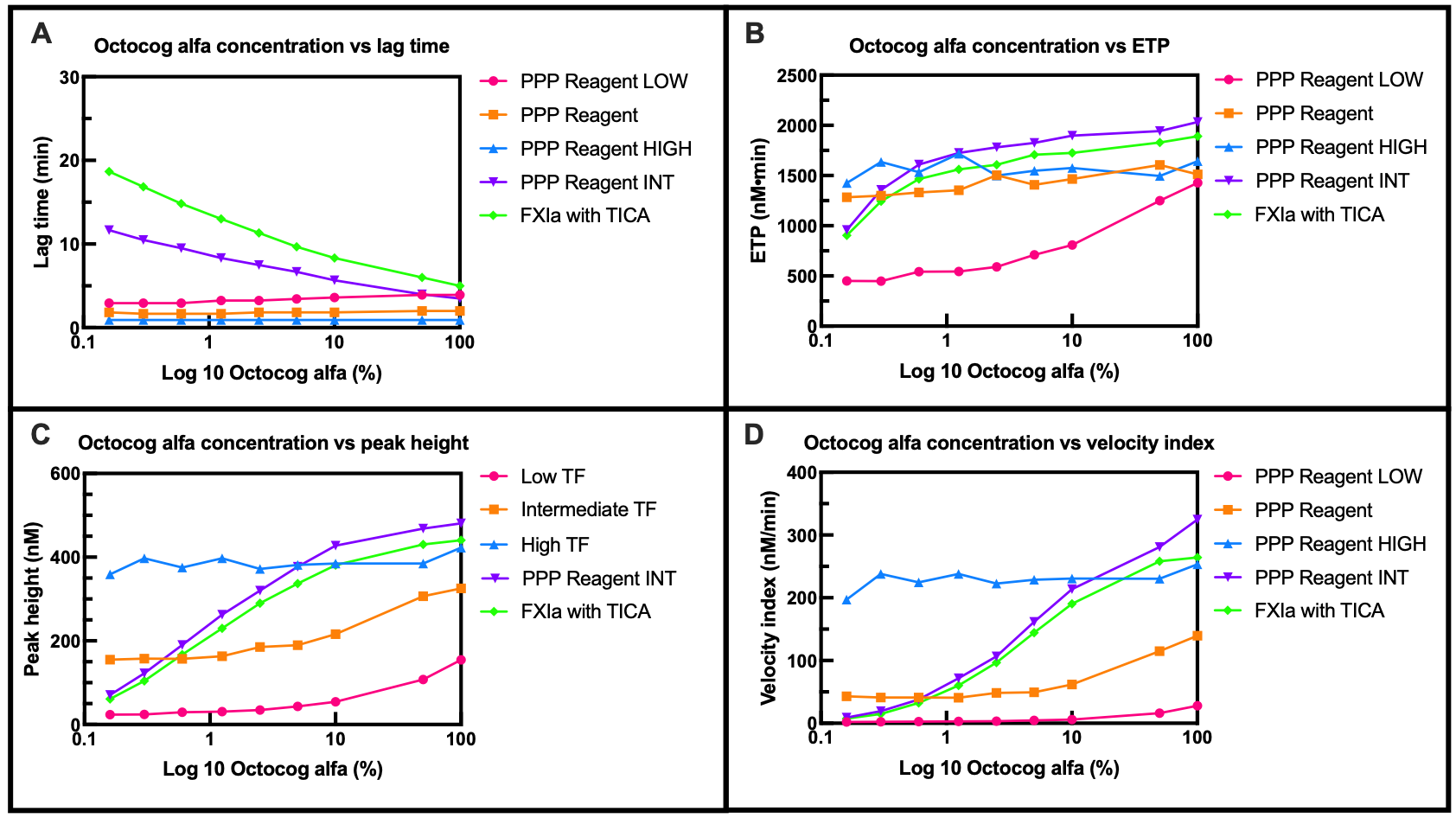


Supplemental figure 3: Octocog alfa level plotted against the lag time (A), ETP (B), peak height (C) or velocity index (D) (derived from thrombin generation measured using PPP Reagent LOW, PPP Reagent, PPP Reagent HIGH, PPP Reagent INT and FXIa with TICA) in FVIII deficient plasma spiked with 0-100% octocog alfa.


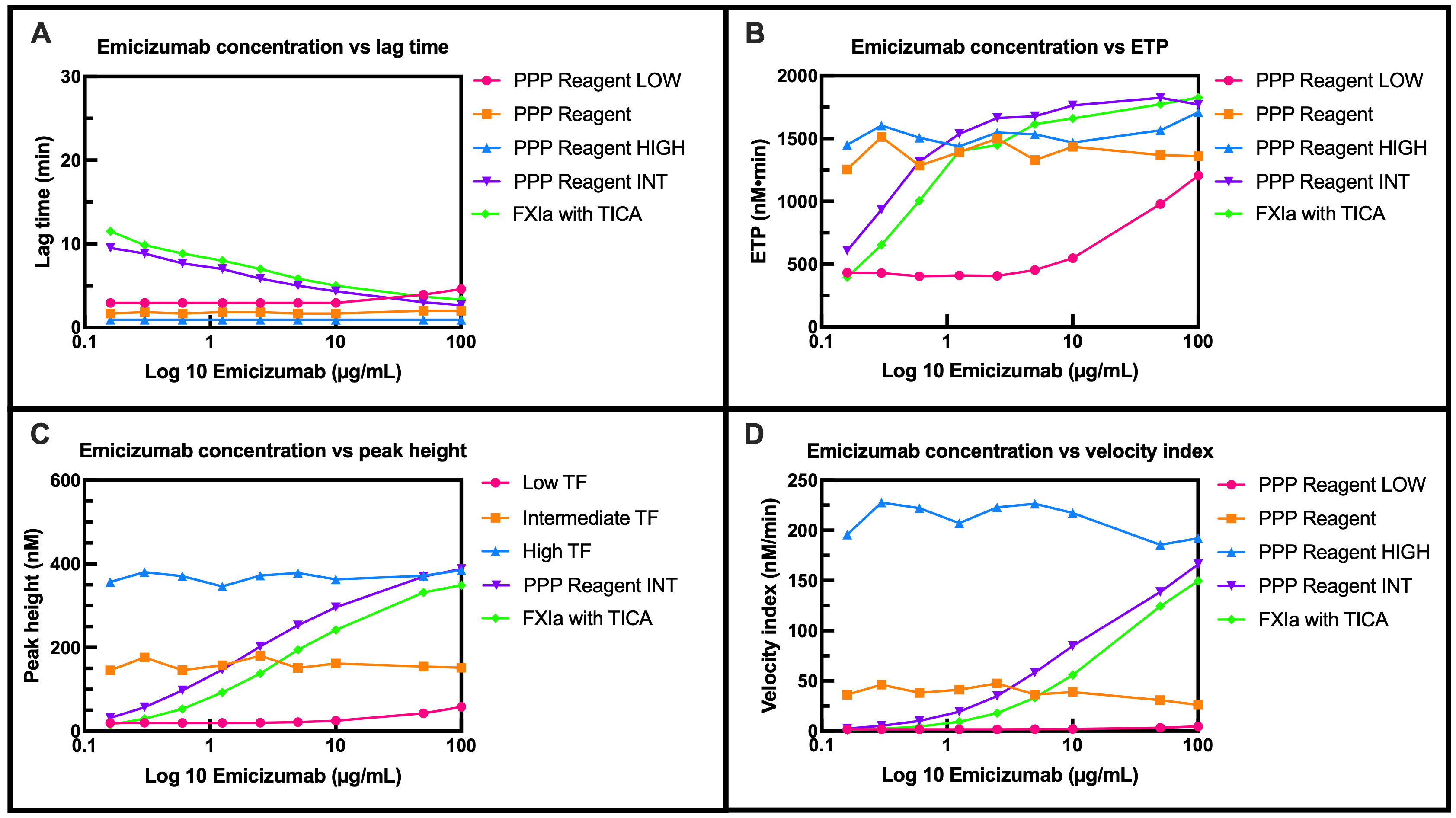


Supplemental figure 4: Emicizumab level plotted against the lag time (A), ETP (B), peak height (C) or velocity index (D) (derived from thrombin generation measured using PPP Reagent LOW, PPP Reagent, PPP Reagent HIGH, PPP Reagent INT and FXIa with TICA) in FVIII deficient plasma spiked with 0-100 µg/mL emicizumab.
